# Development and evaluation of 4WSS electric-driven chassis for high-clearance sprayer

**DOI:** 10.3389/fpls.2023.1258744

**Published:** 2023-09-28

**Authors:** Siwei He, Yue Shen, Yafei Zhang, Hui Liu

**Affiliations:** School of Electrical and Information Engineering, Jiangsu University, Zhenjiang, China

**Keywords:** crop protection, high clearance sprayer, four-wheel self-steering chassis, electrically driven, speed distribution controller

## Abstract

**Introduction:**

The high clearance sprayer with conventional steering mechanisms, as an intelligent spraying machine, is frequently stuck or broken in muddy fields due to the excessive torque load.

**Methods:**

A Four-Wheel Self-Steering (4WSS) electric-driven chassis with a smaller turning radius and better passability is developed to handle complex agricultural terrains. The 4WSS chassis is mainly composed of two custom-designed steering bridges and four in-wheel drive motors. It can achieve steering and driving forward simultaneously through coordinate differential speed control of drive motors, saving a set of dedicated servo steering systems and requiring less torque during steering compared to conventional structures. A kinematic model depicting the speed relationships between four wheels is established via geometric analysis, and a Speed Distribution Controller (SDC) is designed to accomplish locomotion objectives.

**Results:**

Experimental results demonstrate the effectiveness of the new prototype 4WSS chassis system in tracking speed and steering angle. Compared to conventional agricultural chassis, the 4WSS chassis has a smaller turning radius of 2,877 mm.

**Discussion:**

The 4WSS chassis exhibits superior performance in typical field conditions, including muddy terrain, deep gullies, and ridges.

## 1 Introduction

The high-clearance sprayer is an important type of agricultural machinery that aims to protect crops from diseases, insects, and weeds. Its steering performance and obstacle-surmounting performance directly affect working efficiency (Oksanen and Linkolehto, 2013; Ding et al., 2018).

Paddy soil has thixotropic properties. When the sprayer chassis repeatedly walks or turns in the paddy field, the soil structure of the paddy field will be damaged, the bearing capacity and shear capacity will be reduced, and the adhesion will be aggravated (Zeng et al., 2019). This often results in the chassis getting stuck in the mud. It is also easy to cause damage to the chassis transmission system and vertical shaft while struggling (Chen et al., 2020). Zeng et al. (2019) designed a wheel-track compound power chassis for a high-clearance sprayer aimed at preventing excessive sinking in paddy fields. The rear wheel was transformed into a tracked structure, leading to a reduction in its sinking depth. But it will crush more plants when turning. Wang et al. (2017) designed a high-clearance roll-waist multifunctional power chassis aimed at improving the stability of field driving and surmounting ridge performance. However, the muddy situation was not well discussed in that paper. Li et al. (2018) designed a high-clearance self-walking full-hydraulic independent driver for the universal operating chassis. The walking variable pump was directly driven by the engine. Torque was transmitted to the walking system and steering system through a hydraulic pump. Compared with the mechanical transmission chassis, the hydraulic transmission chassis was not only more convenient in layout but also more reliable. It also had a certain effect on the improvement of the ability to surmount obstacles. Actual research on the chassis in the academic community was mostly concentrated on the hydraulic transmission chassis for such advantages (Hui et al., 2018; Li et al., 2019; Liu et al., 2019).

Nevertheless, an electric chassis is more efficient (Park et al., 2019) and causes less pollution (Sato et al., 2022) than a hydraulic chassis, and its proportion in the vehicle field is gradually increasing (Park et al., 2023). Moreover, the electric chassis has better performance on controllability, particularly the four-wheel independent drive (4WID) structure (Wang et al., 2022). Each wheel’s torque or speed can be controlled independently, giving it strong potential in terms of handling stability and flexibility (Liang et al., 2020; Liu et al., 2021).

Based on the 4WID chassis, some academics had further proposed the concept of differential steering, which generated steering torque by separately controlling the speed or torque of each in-wheel motor. Wang et al. (2008) proposed a novel power-assisted differential steering system based on the front two-wheeled differential steering (FTDS) electric vehicle (EV). Then, a vehicle dynamics model with the steering system was built in SIMULINK, and a phase lead compensator was designed to modulate differential torque of the front wheels. Wu et al. (2013); Wu et al. (2014) investigated an FTD EV by analyzing the kinematic model with the Ackermann–Jeantand steering. A speed-following control was designed to achieve electrical differential. Oke et al. (2016) designed a dynamic output feedback controller for an all-wheeled differential steering (AWDS) EV. The vehicle dynamic system with road adhesion was analyzed, and the *H*∞ controller was utilized to improve yaw dynamics performances. Kuslits and Bestle (2019) analyzed the motion of the AWDS EV and proved that differential steering had a comparable steering performance as that of conventional passenger cars through various simulation experiments. This demonstrated that simplifying or omitting the dedicated steering actuators of the vehicle was reasonable. Summing up the above, the methods of differential chassis were mainly based on the kinematic speed-following method or dynamic traction force distribution method. Considering the demand for field chassis and the advantages of electric chassis, the four-wheel self-steering (4WSS) electric chassis was proposed in this paper. Based on the research experience of other differential chassis, the research route was determined to start from kinematics.

Therefore, the main contributions of this paper are as follows: 1) The 4WSS chassis is designed with four independently driven power motors, and the special steering structure improves the steering and surmounting ability in mud; 2) The linkages, designed to constrain the front and rear steering bridge, are analyzed, and the calculation method of its installation point is demonstrated; 3) The 4WSS chassis kinematics model is established, and its SDC is designed; 4) Field tests prove a small steering radius and better passability of the 4WSS chassis.

## 2 Materials and methods

### 2.1 Chassis structure design

**Figure 1:**
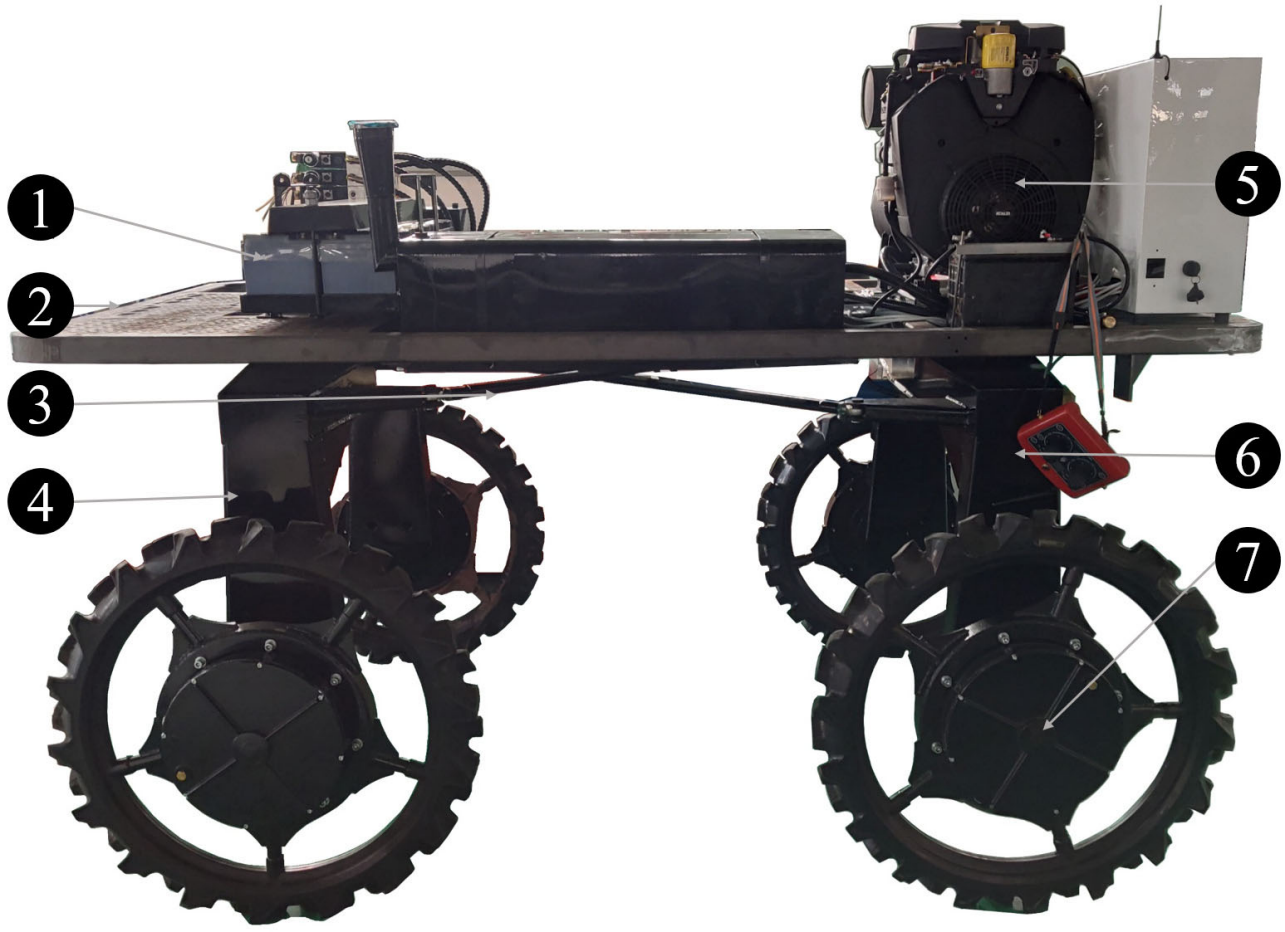
The four-wheel self-steering (4WSS) chassis. 1: lead-acid batteries; 2: frame; 3: linkages; 4: front steering bridge; 5: generator; 6: front steering bridge; 7: wheel (BLDC motor embedded inside).

The actual demand for paddy field machinery is considered for the design of 4WSS chassis, which is sketched in **Figure 1**. The 4WSS chassis is driven independently by in-wheel motors, and the wheels which are embedded Brushless Direct Current Motor (BLDC) motors are installed on both sides of the steering bridges. The steering bridges are both connected to the frame with slewing bearings. So, steering bridges are separately rotated around slewing bearings to form steering angles. The linkages between the front and rear steering bridges make the amplitudes of steering angles equal. Compared with the hydraulic transmission chassis, the electric chassis has advantages of larger torque and stronger capability to surmount obstacles (Ragheb et al., 2013). The 4WSS chassis, steering by controlling four-wheel differentials, is significantly different from the existing differential chassis.

The novel structure of the 4WSS chassis brings challenges to modeling and steering control. And its basic components are divided into three sections: Backbone Structure, Linkage Structure, and Drive System.

#### 2.1.1 Backbone structure

Unlike most existing Ackermann steering chassis, the 4WSS chassis backbone structure mainly consists of three parts: the freely rotating front steering bridge and rear steering bridge and the frame. The front and rear steering bridges are inverted U-shaped structures, and in-wheel motors (wheels with BLDC motors embedded inside) are mounted on both sides. The steering bridges are connected to the frame with slewing bearings.

**Figure 2:**
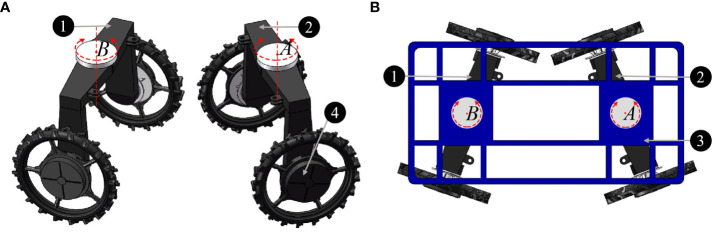
Basic structure of the four-wheel self-steering (4WSS) chassis. **(A)** Front bridge and rear bridge. **(B)** Connection of one main frame and two steering bridges.

The basic structure of the 4WSS chassis is shown in **Figure 2**. As shown in **Figure 2A**, A and B are the center point of the front and rear steering bridge separately. The bridges are connected to the frame with slewing bearings. The assembled figure is shown in **Figure 2B**. The four wheels are named the front left (*fl*) wheel, front right (*fr*) wheel, rear left (*rl*) wheel, and rear right (*rr*) wheel according to their positions on the chassis.

**Figure 3:**
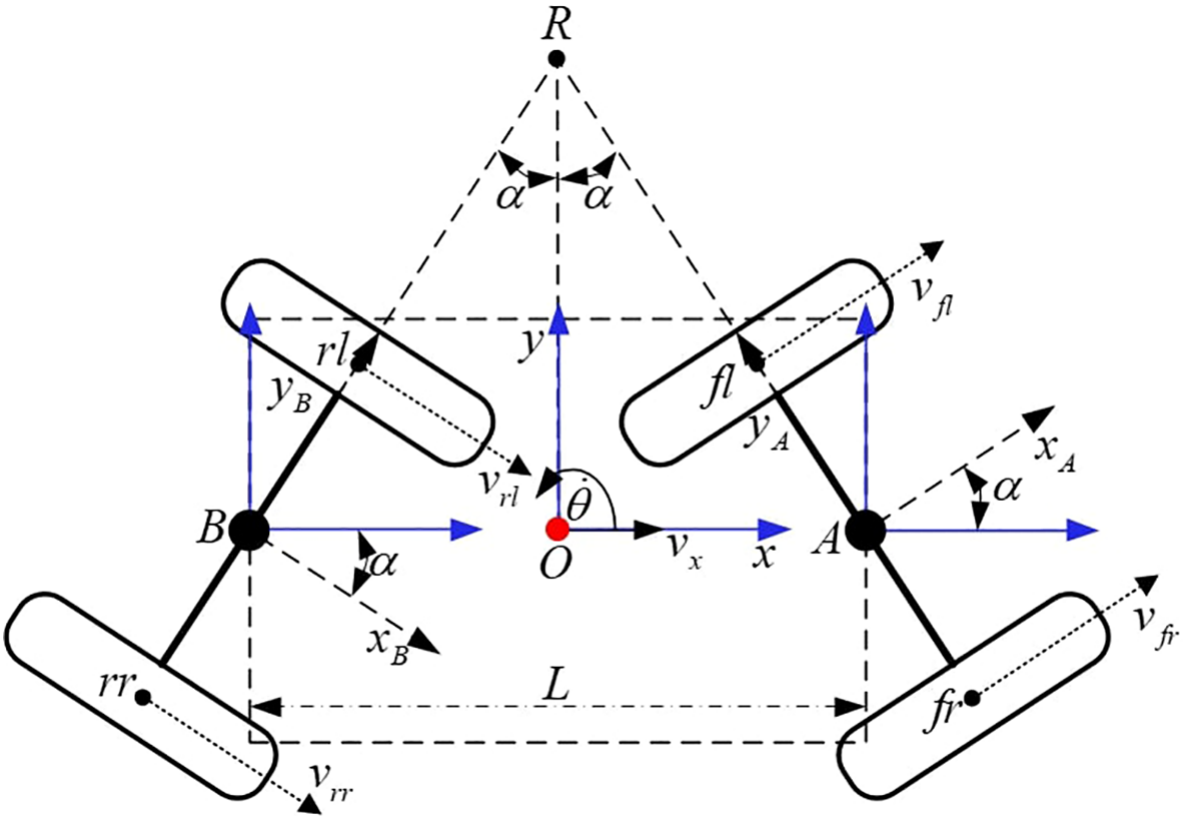
Self-steering structure diagram.

To better demonstrate the working principle of the chassis, the three Cartesian coordinate systems are established as sketched in **Figure 3**. At the geometric center of the frame, the coordinate system *O* is attached. Axis *x* is parallel to *OA* and points to *A*. At the geometric center of the front steering bridge, the coordinate system *A* is attached. Axis *y_A_* is parallel to the bridge and points to the front left wheel. In the same way, at the geometric center of the rear steering bridge, the coordinate system *B* is attached. Axis *y_B_* is parallel to the bridge and points to the rear left wheel. Take the front steering bridge as an example, define the front steering angle as the angle rotated from *x* to *x_A_* and the anticlockwise direction represents positive. Four in-wheel motors are mounted on the front and rear steering bridges, and each motor can be driven independently.

The chassis runs straight when the front and rear steering angles are 0. In the case that the front steering angle is greater than the rear steering angle, the chassis will turn left and vice versa. The front and rear steering angles can be changed by the speed of the corresponding in-wheel motors. So, the critical problem of controlling this chassis is controlling the speed of the four in-wheel motors.

#### 2.1.2 Linkage structure

The linkage structure is designed to improve the stability and anti-disturbance ability of the chassis by formulating hard constraints on steering angles of the front and rear bridges. With such constraint, the steering angles are consistently kept opposite and numerically equal.

**Figure 4:**
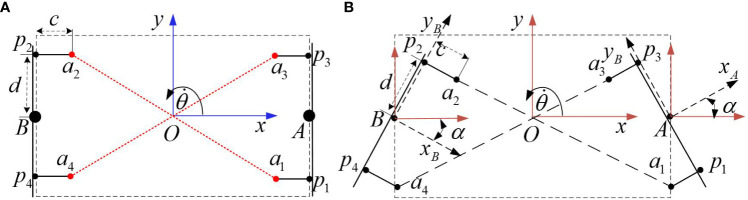
Linkage structure diagram. **(A)** The straight run state. **(B)** The steering run state.

**Figure 4** is the linkage structure diagram. *a*_1_ ∼ *a*_4_ are the four installation points of the linkage structure. The two linkages *a*_1_ *a*_2_ and *a*_3_ *a*_4_ are cross-connected at four connecting points. The major challenge of the linkage structure lies in the position selection of the linkage installation points *a*_1_ ∼ *a*_4_. The points *p*_1_ ∼ *p*_4_ are on the center line of the steering bridge respectively, and the distance from the center of the steering bridge is *d*. The distance between point *a_i_* and point *p_i_* is *c*(*i* = 1 ∼ 4). The line *p_i_a_i_* is perpendicular to axis *y_B_* or *y_A_*.

The straight run state is shown in **Figure 4A**, and the steering run state is shown in **Figure 4B**. The optimal installation points, the positions of *a*_1_ to *a*_4_, should be settled where the two linkages remain the same length throughout the steering process. Only one linkage will be analyzed by considering the symmetrical property. With regard to the length between *a*_1_ and *a*_2_, when the steering bridge angle is *α*, the length of the linkage is:


(1) $$H\;\left(\alpha \right)\;=\sqrt{4d^{2}cos^{2}\left(\alpha \right)+{\left(L-\;2c\;cos\left(\alpha \right)\right)}^{2}}$$

The linkage steering error *E* (*α*) is defined as the difference between *H* (0) and *H* (*α*).


(2) $$\begin{array}{c} E\;\left(\alpha \right)=H\;\left(0\right)-H\;\left(\alpha \right)=\sqrt{4d^{2}+{\left(L-2c\right)}^{2}}\\ -\sqrt{4d^{2}\cos{\;}^{2}\;(\alpha )+{\left(L-2c\cos\;\left(\alpha \right)\right)}^{2}} \end{array}$$

When the chassis is moving, the range of the steering angle should be limited. The maximum steering angle is called the mechanics-limited steering angle, and the value is 25°. As the steering angle increases, the stability progressively decreases. The mechanics-limited steering angle is α∈[−25°,25°] . Since *E*(*a*) is an even function, it is just analyzed when α∈[0°,25°] . The derivative of *E* (*α*) is:


(3) $$\begin{array}{l} \dot{E}\;\left(\alpha \right)=\frac{4d^{2}\sin\;\left(\alpha \right)\cos\;\left(\alpha \right)}{\sqrt{4d^{2}\cos{\;}^{2}\left(\alpha \right)+{\left(L-2c\cos\;\left(\alpha \right)\right)}^{2}}}\\ -\frac{2c\;\left(L-2c\cos\;\left(\alpha \right)\right)\;\mathrm{son}\;\left(\alpha \right)}{\sqrt{4d^{2}\cos{\;}^{2}\left(\alpha \right)+{\left(L-2c\cos\;\left(\alpha \right)\right)}^{2}}} \end{array}$$

An optimal installation point d and c can be solved from Equation 4, which implies E (α) = E (0) = 0 under such solution.


(4) $$\dot{E}\left(\alpha \right)=0$$

And the solution of Equation 4 is


(5) $$\{\begin{array}{c} d=0\\ c=0 \end{array}$$

This is not a feasible installation point. So, there is no perfect installation point to keep the linkage remaining in the same length at all steering angles. Thus, a linkage with a slightly deformable capability is taken into consideration.

When handling the position of the installation point, *d* is selected first, determined by the chassis manufacturer. Then, an appropriate *c* can be obtained by fixing the steering angles *α* and *d*, and the results can be verified at last.

Solve Equation 6 to achieve a suitable *c*.


(6) E(α)=0

The two solutions to *c* are


(7) $$c_{1}=\frac{L-\sqrt{\left(L-2d\cos\;\left(\alpha \right)-2d\right)\left(L+2d\cos\;\left(\alpha \right)+2d\right)}}{2\left(\cos\;\left(\alpha \right)+1\right)}$$

(8) $$c_{1}=\frac{L+\sqrt{\left(L-2d\cos\;\left(\alpha \right)-2d\right)\left(L+2d\cos\;\left(\alpha \right)+2d\right)}}{2\;\left(\cos\;\left(\alpha \right)+1\right)}$$

Design the parameters *L* = 1.7 m, *d* = 0.406 m, *α* = 25°. Then, the solutions can be solved from Equations 7 and 8 that


(9) $$\{\begin{array}{c} c_{1}=0.262\\ c_{2}=0.630 \end{array}$$

**Figure 5:**
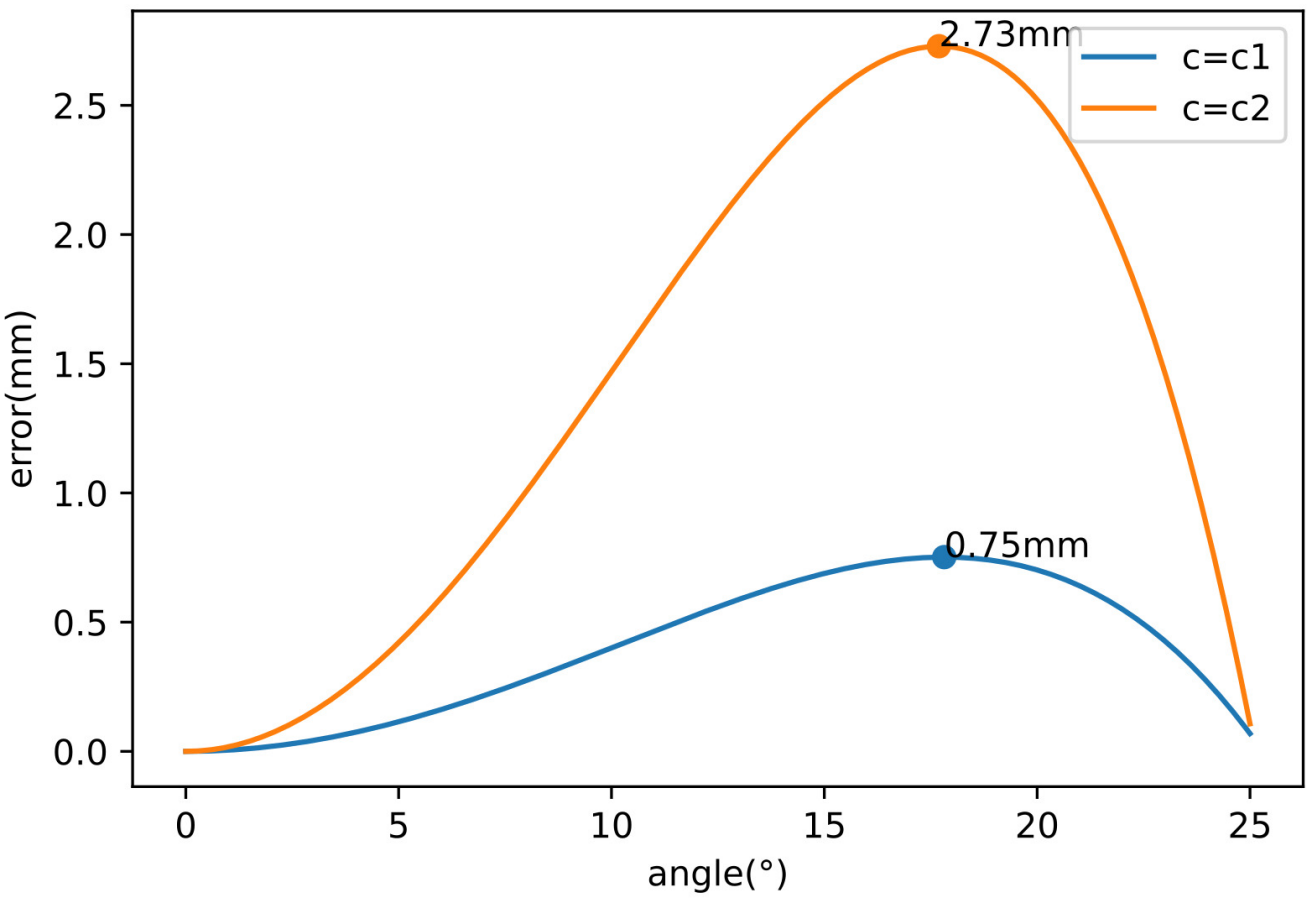
Error graph with different values about c.

Use the calculated *c*_1_ and *c*_2_ to plot the error graphic (**Figure 5**) of *E* (*α*) with respect to *α*.

The corresponding maximum error varies as *c* changes. When *c* = *c*_1_, the maximum error is 0.75 mm, and when *c* = *c*_2_, the maximum error is 2.73 mm. So *c*_1_ is a suitable solution. The linkage remains deformation during steering, which is less than 0.75 mm within the elastic range.

#### 2.1.3 Drive system

Four in-wheel motors mounted on the steering bridges provide tractive force. Therefore, the design of the drive system is mainly based on the power distribution system, the in-wheel motor and driver system, and the drive control system.

##### 2.1.3.1 Power distribution system

**Figure 6:**
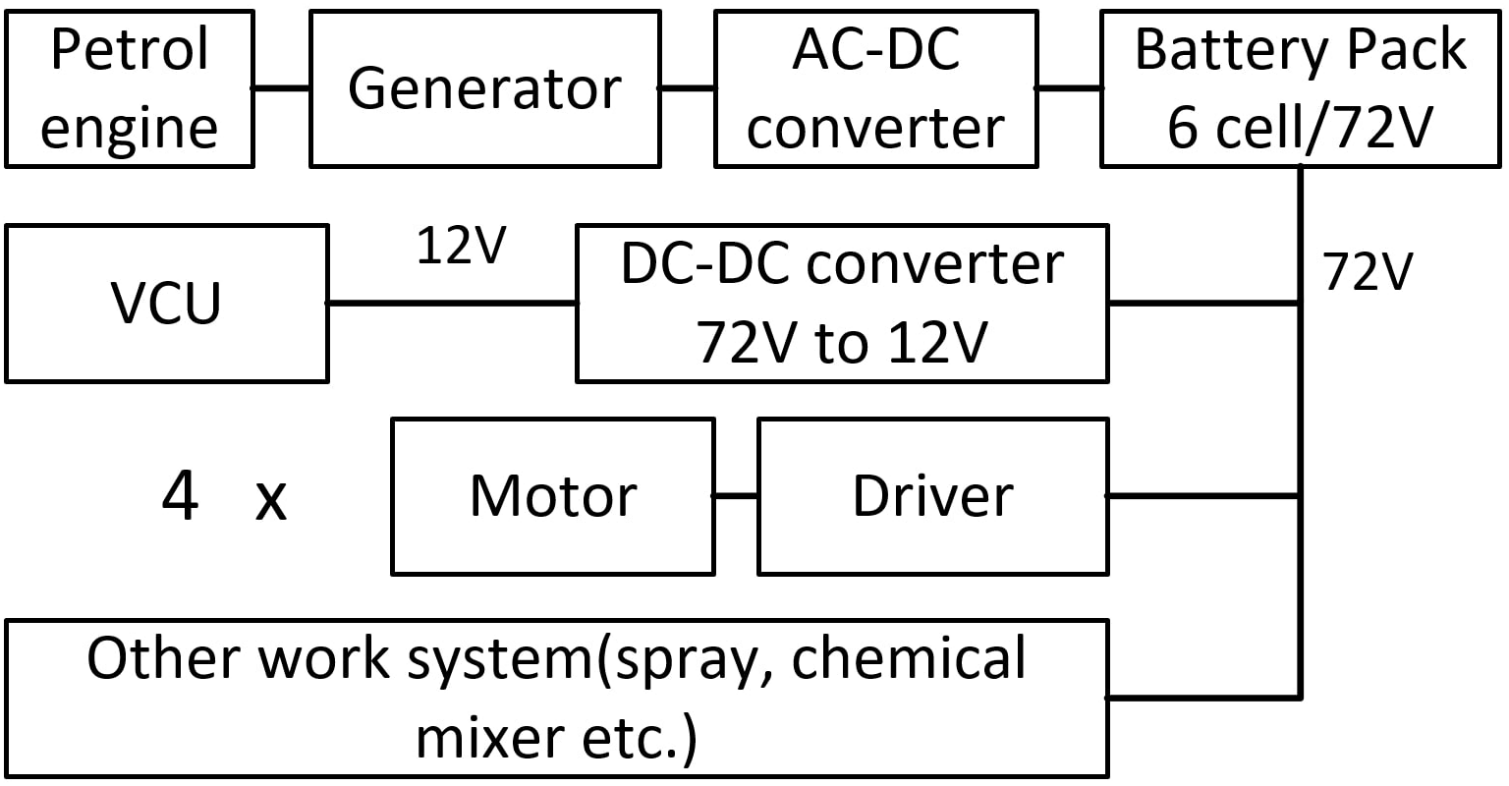
Power distribution system diagram.

For the chassis to function smoothly, a stable power distribution system is essential, which is sketched in **Figure 6**. The electrical bus connects the batteries and generator to the drivers of the motors, DC-DC converter, and other work systems. One battery pack with six cells is attached to the front of the chassis. Each battery provides a nominal voltage of 12 V, and six batteries together proved a nominal bus voltage of 72 V. A power generation unit, composed of a petrol engine, generator, and AC-DC converter, is housed at the rear of the chassis. There are mainly three kinds of electrical equipment on the chassis: VCU, motor drivers, and other work systems (spray, chemical mixer, etc.). VCU draws 12 V of direct electric current from the DC-DC converter, while the motor drivers and other working systems are connected to the 72-V bus directly.

##### 2.1.3.2 The in-wheel motor and driver system

**Figure 7:**
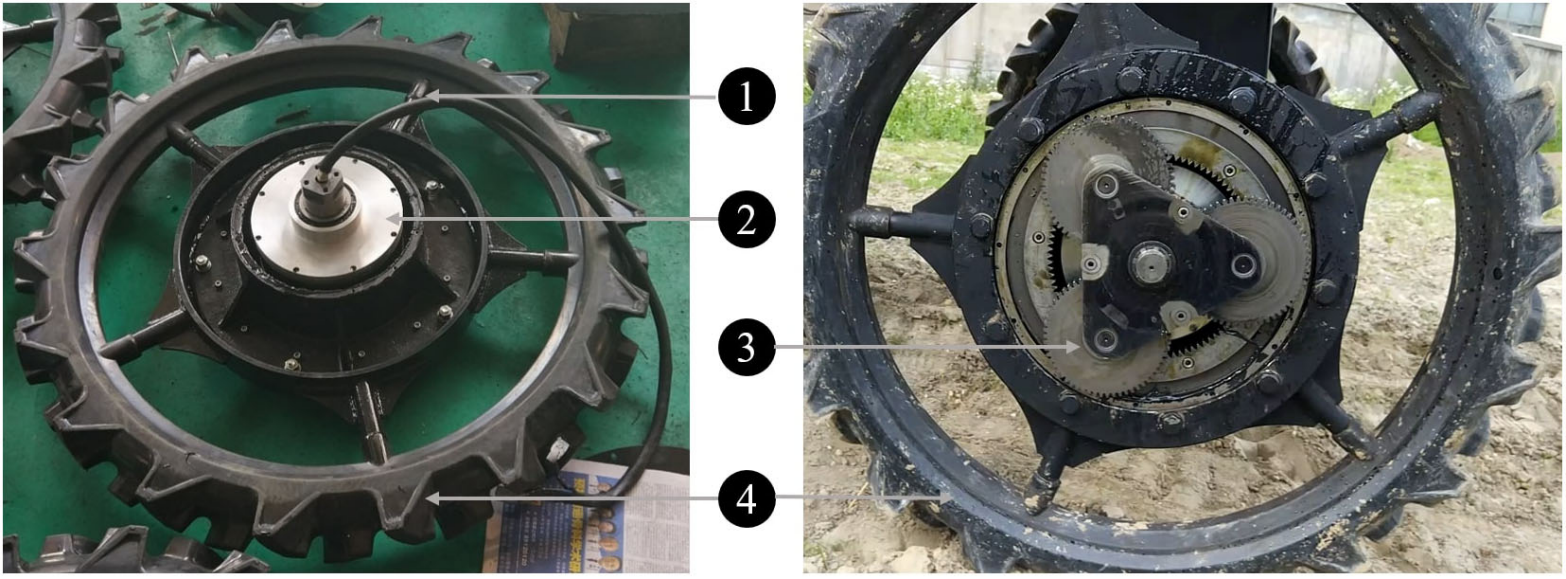
The wheel of the four-wheel self-steering (4WSS) chassis. 1: hub; 2: embedded permanent magnet synchronous motor; 3: reduction mechanism; 4: solid rubber tire.

As shown in **Figure 7**, an in-wheel motor mainly consists of four parts: a hub, an embedded permanent magnet synchronous motor (BLDC), a reduction mechanism, and a tire. Five spokes are used between the rim and the center, so the wheel is mostly hollow. The wheels do not use conventional pneumatic tires but solid rubber tires. Solid rubber tires require less maintenance and can be made with a deeper tread for better grip in muddy paddy fields. The tire width is 11 cm, and a wider tire has a larger contact area with the road surface, making movement easier in muddy fields.

The embedded motor has a rated power of 4 kW, a rated voltage of 72 V, and a rated speed of 1,000 rpm. The in-wheel motor is composed of a planetary gear coaxial deceleration structure with a reduction ratio of 1:19, which reduces the speed of the in-wheel motor to 52.6 rpm. The wheel radius is 0.483 m, so the rated chassis speed of the in-wheel motor is near 10 kmh^−1^.

The motor driver (Kelly QSKLS8430H) is selected for driving the embedded motor. It has a rated current of 100 A and an input voltage of 24 V~105 V. The driver adjusts the input voltage of the BLDC motor, according to the control signal from the VCU, to control the speed of the wheel. The CAN bus is used between the driver and the controller.

Kelly QSKLS8430H is a general-purpose EV driver, its basic functions including torque output and regenerative braking. The driver will actively ignore the given reverse torque output when the motor speed is positive. Therefore, in this case, it needs to be set to the regenerative braking mode, so that the reverse torque is small but sufficient for speed control.

##### 2.1.3.3 Drive control system

**Figure 8:**
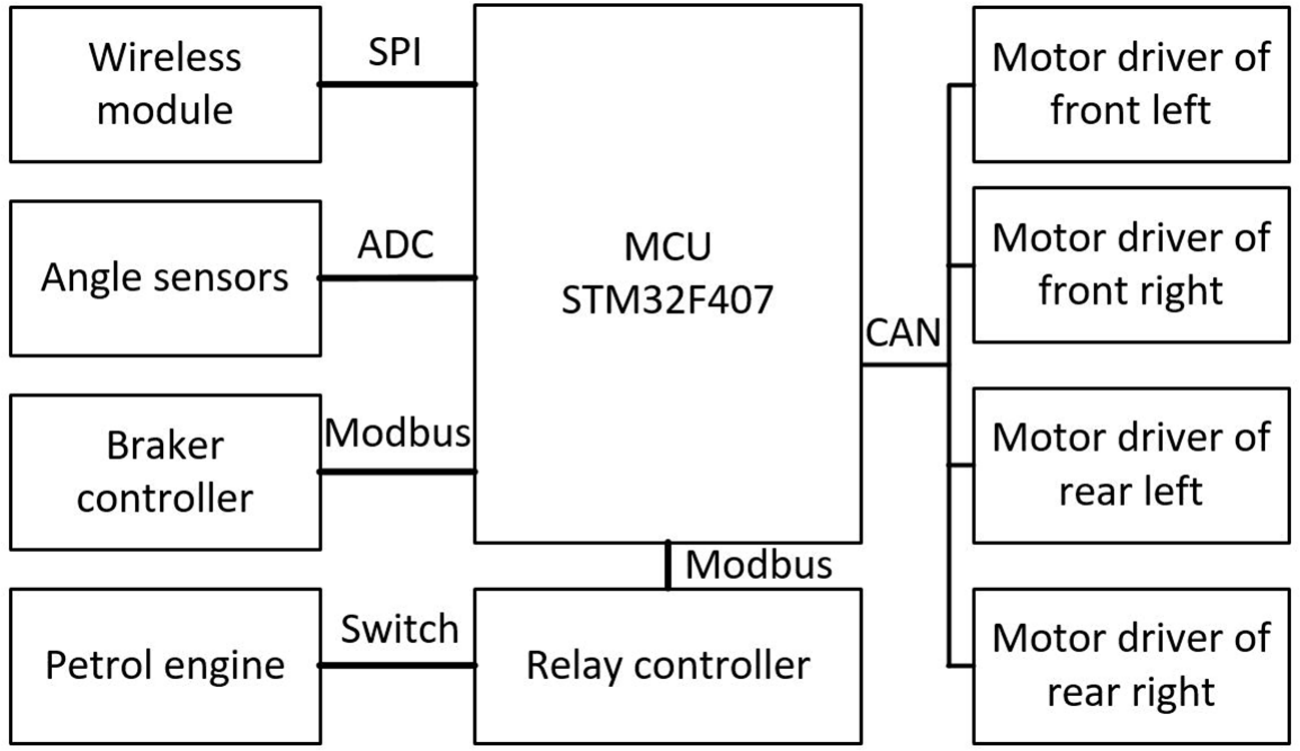
Communication-level system diagram.

**Figure 8** depicts a diagram of the communication-level system. The platform is controlled by a high-performance microcontroller unit (MCU) STM32F407VET6. This MCU communicates with four motor drivers via the CAN bus interface. The drivers receive the control message from the MCU, and its output wheel velocity gives feedback to the MCU. The wireless module, the motor drive system, and the angle sensors constitute the minimum system of chassis operation. The wireless module communicates with the remote controller to receive commands from the operator. Two linear hall angle sensors are mounted on the front and the rear steering bridge, respectively. Therefore, the front and rear steering angles can be obtained in real time through the 12-bit ADC of the MCU. The MCU handles control tasks by assembling the operator’s commands and the feedback signals. The in-wheel motors are braked via the power-off of the brake coils, which is controlled by the brake controller. This can be used when braking or parking. The brake controller communicates with the MCU via Modbus, receiving MCU control instructions and returning the error of the brake system. The relay controller mainly controls the operation of the generator and other operating systems.

#### 2.1.4 Structural comparison and challenges

**Figure 9:**
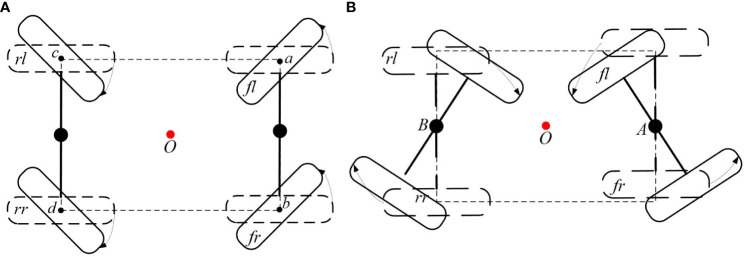
Comparison of different steering structures. **(A)** Conventional four-wheel independent drive (4WID) structure with dedicated steering actuators. **(B)** The four-wheel self-steering (4WSS) utilizes only drive motors without steering actuators.

Controlling movement and steering of vehicles are two major functions of the chassis. For conventional 4WID chassis, movement is manipulated by drive motors directly and steering is handled by steering motors separately. As shown in **Figure 9A**, the wheels of the conventional 4WID structure rotate around their center points. The steering of the chassis is restricted by the deflection angle of each wheel. Each wheel rotates around its center point, and the mounting shaft directly bears the reverse torque when steering. When the steering mechanism gets deep sinking in the mud, the wheel has to push the side mud directly away to steer. Under such circumstances, the chassis often fails to provide sufficient torque to turn, or the steering structure cannot withstand excessive torque, resulting in damage.

Different from the conventional 4WID chassis as described above, each wheel of the novel 4WSS chassis rotates around the centers of its corresponding steering bridge, as shown in **Figure 9B**. The front bridge, taken as an example, when the differential speed of *fl* wheel and *fr* wheel exists, rotates around the mounted point *A*, completely motivated by in-wheel motors. It is not necessary to completely push the side mud during steering. Consequently, the 4WSS chassis requires less steering torque, provides the longer force arm, and replaces the dedicated steering motors with more powerful in-wheel motors. The 4WID 4WSS structure has the potential to get better passability in dealing with muddy conditions.

The conventional 4WID chassis requires dedicated steering servo motors to steer, while the 4WSS chassis employs the differential speed of the in-wheel motors to steer. The 4WSS chassis saves four steering servo motors, reducing the complexity and cost of the hardware system. With the comparison analysis, the structure of the 4WSS chassis is simpler to make higher reliability. On the other hand, challenges in designing the controller arise from the coupling between the in-wheel motors, which completely control the action of the chassis.

### 2.2 Kinematic model build

The characteristics of the 4WSS chassis indicate its difference in nature from the Ackermann steering structure. The movement of the 4WSS chassis is based on the four-wheel differential speed. Therefore, the study of the relationship between the wheel speed and the chassis movement is the basis for designing the control system.

**Figure 3** depicts a diagram of the self-steering structure. On the premise of linkage constraint as described in the section *Linkage Structure*, it can be considered that the front and rear steering angle amplitudes are equal. The prolongation lines of the front and rear steering bridge meet at point *R*. According to geometric relations, ∠*BRO* = ∠*AOR* = *α* can be obtained. Assuming no skidding between the in-wheel motors and the ground, point *R* is the steering center of the 4WSS chassis.

*v_fl_*, *v_fr_*, *v_rl_*, and *v_rr_* represent the corresponding speed of each wheel. *v_x_* is 4WSS chassis speed. *L* is the wheelbase, and *α* is the steering angle. The radius of the steering bridge *I* and the radius of the 4WSS chassis *K* can be obtained.


(10) $$I=RA=\frac{L}{2\sin\;\alpha }$$

(11) $$K=RO=\frac{L}{2\tan\;\alpha }$$

*D* is wheel track, and *θ* ^˙^ is yaw rate. In the case of no slippage, the relationship between the yaw rate and the speed of the four in-wheel motors can be obtained in Equation 12.


(12) $$\dot{\theta }=\frac{v_{fl}}{I-\frac{D}{2}}=\frac{v_{fr}}{I+\frac{D}{2}}=\frac{v_{rl}}{I-\frac{D}{2}}=\frac{v_{rr}}{I+\frac{D}{2}}$$

Rewriting Equation 12 yields the relationship between the in-wheel motor speed and the yaw rate.


(13) $$\dot{\theta }=\frac{v_{fl}+v_{fr}+v_{rl}+v_{rr}}{4I}$$

According to the kinematic relationship, the speed of the chassis *v_x_* can be written as Equation 14.


(14) $$v_{x}=\dot{\theta }k$$

Based on Equations 10–14, Equation 15 can be derived.


(15) $$v_{x}=\frac{v_{fl}+v_{fr}+v_{rl}+v_{rr}}{4}\cos\;\alpha$$

Generally, α∈[−25°,25°] can be approximated as cos*α* = 1. Thus, the approximated chassis speed meets Equation 16.


(16) $$v_{x}=\frac{v_{fl}+v_{fr}+v_{rl}+v_{rr}}{4}$$

According to Equations 13 and 16, the result can be derived.


(17) $$\dot{\theta }=\frac{v_{x}}{I}$$

The speed of each in-wheel motor can be decomposed of the speed around the chassis steering center *R* and the speed around the bridge steering center *A* or *B*.

The velocities of the four in-wheel motors can be obtained as shown in Equations 18–21.


(18) $$v_{fl}=\dot{\theta }\left(I-\frac{D}{2}\right)-\dot{\alpha }\frac{D}{2}$$

(19) $$v_{fr}=\dot{\theta }\left(I+\frac{D}{2}\right)+\dot{\alpha }\frac{D}{2}$$

(20) $$v_{rl}=\dot{\theta }\left(I-\frac{D}{2}\right)+\dot{\alpha }\frac{D}{2}$$

(21) $$v_{rr}=\dot{\theta }\left(I+\frac{D}{2}\right)-\dot{\alpha }\frac{D}{2}$$

Substitute Equation 17 into Equations 18–21, replace *θ* ^˙^ with *v_x_*, and then obtain Equations 22–25.


(22) $$v_{fl}=v_{x}\left(1-\frac{D}{2I}\right)-\dot{\alpha }\frac{D}{2}$$

(23) $$v_{fr}=v_{x}\left(1+\frac{D}{2I}\right)+\dot{\alpha }\frac{D}{2}$$

(24) $$v_{rl}=v_{x}\left(1-\frac{D}{2I}\right)+\dot{\alpha }\frac{D}{2}$$

(25) $$v_{rr}=v_{x}\left(1+\frac{D}{2I}\right)-\dot{\alpha }\frac{D}{2}$$

The theoretical model of the 4WSS chassis reveals the correlation between the chassis speed, the steering angle, and the speed of the four wheels. Chassis speed, composed of the in-wheel motor speed and steering angular velocity, is restricted by the limited in-wheel motor speed.

According to the principle of differential steering, the speed of the in-wheel motors on the inner and outer sides varies during steering. It must be ensured that the speed of each of the four wheels shall not be greater than the maximum speed *V* of the in-wheel motor, as shown in Equation 26.


(26) $$\{\begin{array}{l} \left|v_{fl}\right|\leq V\\ \left|v_{fr}\right|\leq V\\ \left|v_{rl}\right|\leq V\\ \left|v_{rr}\right|\leq V \end{array}$$

Union of Equations 22–25 can get Equation 27.


(27) $$\left|v_{x}\right|\leq \frac{\left|VL-\left|\dot{\alpha }\right|\frac{DL}{2}\right|}{L+D\left|\sin\;\alpha \right|}$$

To ensure stability during walking, the maximum steering angular speed of the steering bridge will be limited during operation. Let |*α*˙| ≤ *α*˙*_max_*, and rewrite Equation 27 to get Equation 28.


(28) $$\left|v_{x,max}\right|=\frac{\left|VL-{\dot{\alpha }}_{max}\frac{DL}{2}\right|}{L+D\left|\sin\;\alpha \right|}$$

It can be seen that the maximum speed during the operation of the chassis is limited by the maximum speed of the in-wheel motor, the steering angle, and the steering angular speed. Once the maximum speed of the in-wheel motor is determined, the maximum speed of the chassis decreases as the steering angle increases.

Assuming that the chassis is in a steady state and turned at its maximum steering angle. That is *V* = 10 kmh^−1^ (\dot{\alpha}{max}=0) = 2.78 ms^−1^, *α* = 24° and parameter of the 4WSS chassis is *L* = 1.7 m, *D* = 1.5 m. To avoid the situation where the mechanics-limited steering angle cannot be reached due to sensor errors and mechanical manufacturing errors, *α* is set to 24°. The value is called program-limited steering angle. The maximum speed of the chassis in the steady state is


(29) $$\left|v_{x,max}\right|\;=\;2.0\;ms^{-1}$$

According to Equations 28 and 29, when the steering angle is 24°, the speed of the chassis cannot exceed 2.0 ms^−1^. And speed cannot exceed 2.8 ms^−1^ when going straight.

### 2.3 Speed Distribution controller design

The 4WSS chassis bases its action on target speed and steering angle from the remote control. Set the target steering angle positive or negative to make the chassis turn left or right, while zero to make it go straight. The essence of the 4WSS chassis control system is a servo system that follows the target speed and steering angle from the remote control.

The key to 4WSS chassis motion control is to properly distribute the speeds of the four in-wheel motors according to the control objectives. The correlation between the speed of the four in-wheel motors, the speed of the chassis, and the steering angle can be obtained from the section *Kinematic Model Build*. According to Equations 22–25, the expression about in-wheel motor speeds with the target speed can be directly obtained.


(30) $${\bar{v}}_{fl}=v_{T}\left(1-\frac{D}{2I}\right)-{\dot{\alpha }}_{p}\frac{D}{2}$$

(31) $${\bar{v}}_{fr}=v_{T}\left(1+\frac{D}{2I}\right)+{\dot{\alpha }}_{p}\frac{D}{2}$$

(32) $${\bar{v}}_{rl}=v_{T}\left(1-\frac{D}{2I}\right)+{\dot{\alpha }}_{p}\frac{D}{2}$$

(33) $${\bar{v}}_{rr}=v_{T}\left(1+\frac{D}{2I}\right)-{\dot{\alpha }}_{p}\frac{D}{2}$$

where *V_T_* is the target speed, and ${\dot{\alpha }}_{p}$ is the steering angular velocity. ${\bar{v}}_{fl}$ , ${\bar{v}}_{fr}$ , ${\bar{v}}_{rl}$ , and ${\bar{v}}_{rr}$ are the target speeds of the corresponding in-wheel motors.

The steering angle error is *α_T_* − *α_s_*. When it is positive, ${\dot{\alpha }}_{p}$ should be made positive to lower such error, and vice versa. So, a *P* controller, where *α_s_* is the feedback signal, can be designed.


(34) $${\dot{\alpha }}_{p}=sat\;\left(k\left(\alpha_{T}-\alpha_{s}\right),{\dot{\alpha }}_{max}\right)$$

where *k* is the *P* controller parameter, *α_T_* is the target steering angle, *α_s_* is the actual steering angle, and *α*˙*_max_* is the maximum output limit of the controller.

The function *sat* (*x,M*) is a saturation function. And its definition is shown in Equation 35. It is mainly to limit the maximum steering angular velocity to prevent instability caused by excessive steering angle deviation.


(35) $$sat\;\left(x,M\right)=\{\begin{array}{c} M,\left(x\geq M\right)\\ x,\left(-M<x<M\right)\\ -M,\left(x\leq -M\right) \end{array}$$

To ensure that the in-wheel motor can have sufficient speed margin to deal with disturbances, the maximum speed of the 4WSS chassis needs to be limited in real time. The target speed should be limited according to Equation 36.


(36) $$v_{T}=sat\;(v_{user},v_{x,max})$$

where *v_user_* is the target value directly given by the user via remote control.

Since the motor driver does not assemble the speed servo function, a *PID* controller is selected for the speed servo inner loop (Mohanraj et al., 2022). The difference is that, for the Kelly QSKLS8430H, the speed servo controller needs to truncate the reverse output and set the driver to brake mode.

## 3 Testing

**Figure 10:**
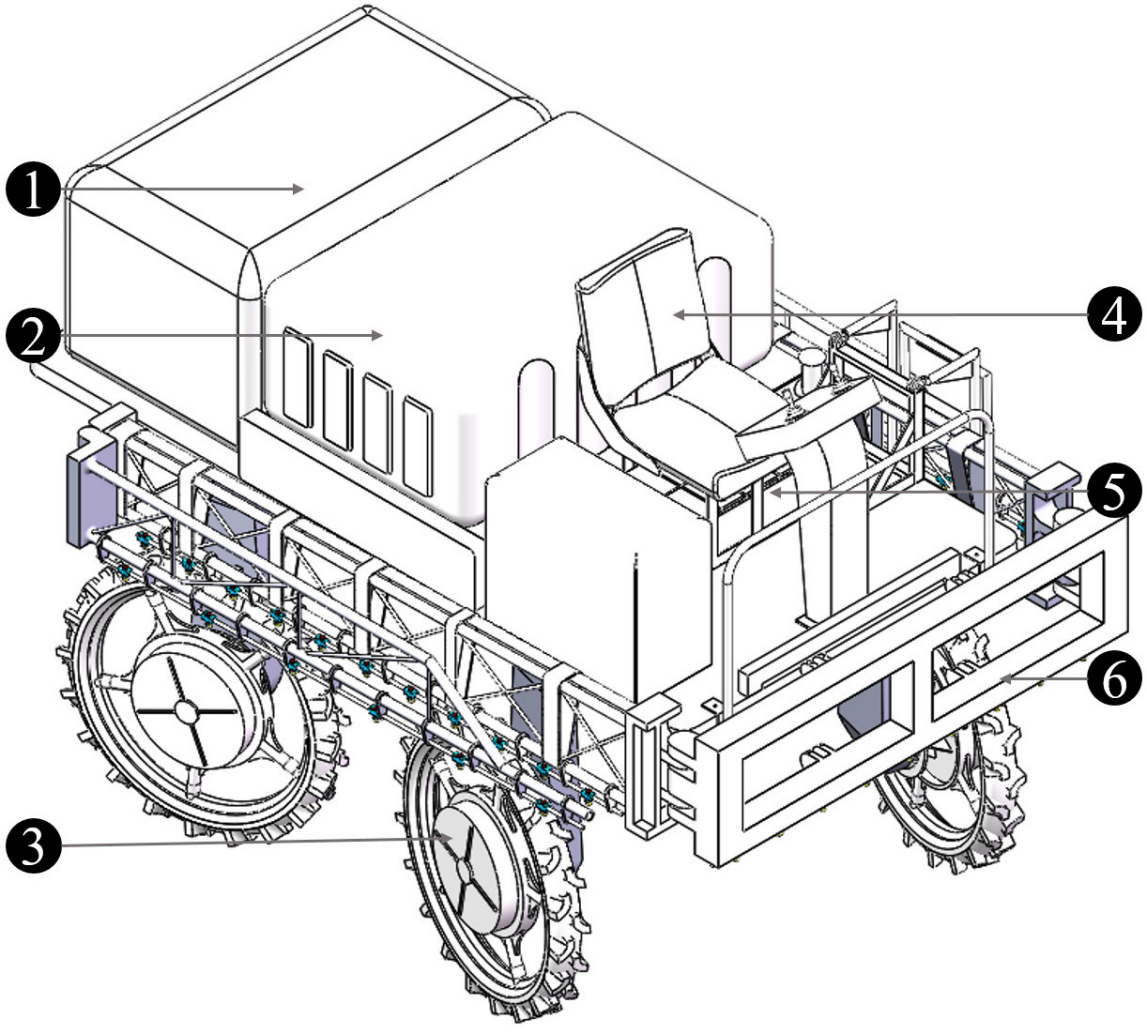
The four-wheel self-steering (4WSS) chassis with spraying system. 1: dust guard (petrol engine, generator, and water pump inside); 2: water tank; 3: wheel; 4: seat; 5: battery pack; 6: spray boom.

The 4WSS chassis with a spraying system is sketched in **Figure 10**. The chassis weighs 1,380 kg, with a wheelbase of 1.5 m, ground clearance of 1.1 m, and overall dimensions of 3,680 × 1,720 × 2,130 mm^3^. The water tank, spray boom, and water pump required for the spraying system are mounted on the chassis. The 500-L water tank is located in the middle of the chassis. The generator and water pump are installed on the rear of the chassis. These components are protected by dust covers, which prevent dust from getting into the machine and avoid accidental touch by the operator. The battery pack is mounted on the front, and the seat is located above the battery pack. The spray arm is mounted on the chassis head. It unfolds to 12 m and folds on both sides when not in operation.

Experimental tests were conducted to evaluate the kinematic performance of the 4WSS chassis and the effectiveness of the kinematic-based control algorithms. A section of a flat road and a piece of muddy field were chosen as representative scenes.

### 3.1 Control of speed and steering angle

**Figure 11:**
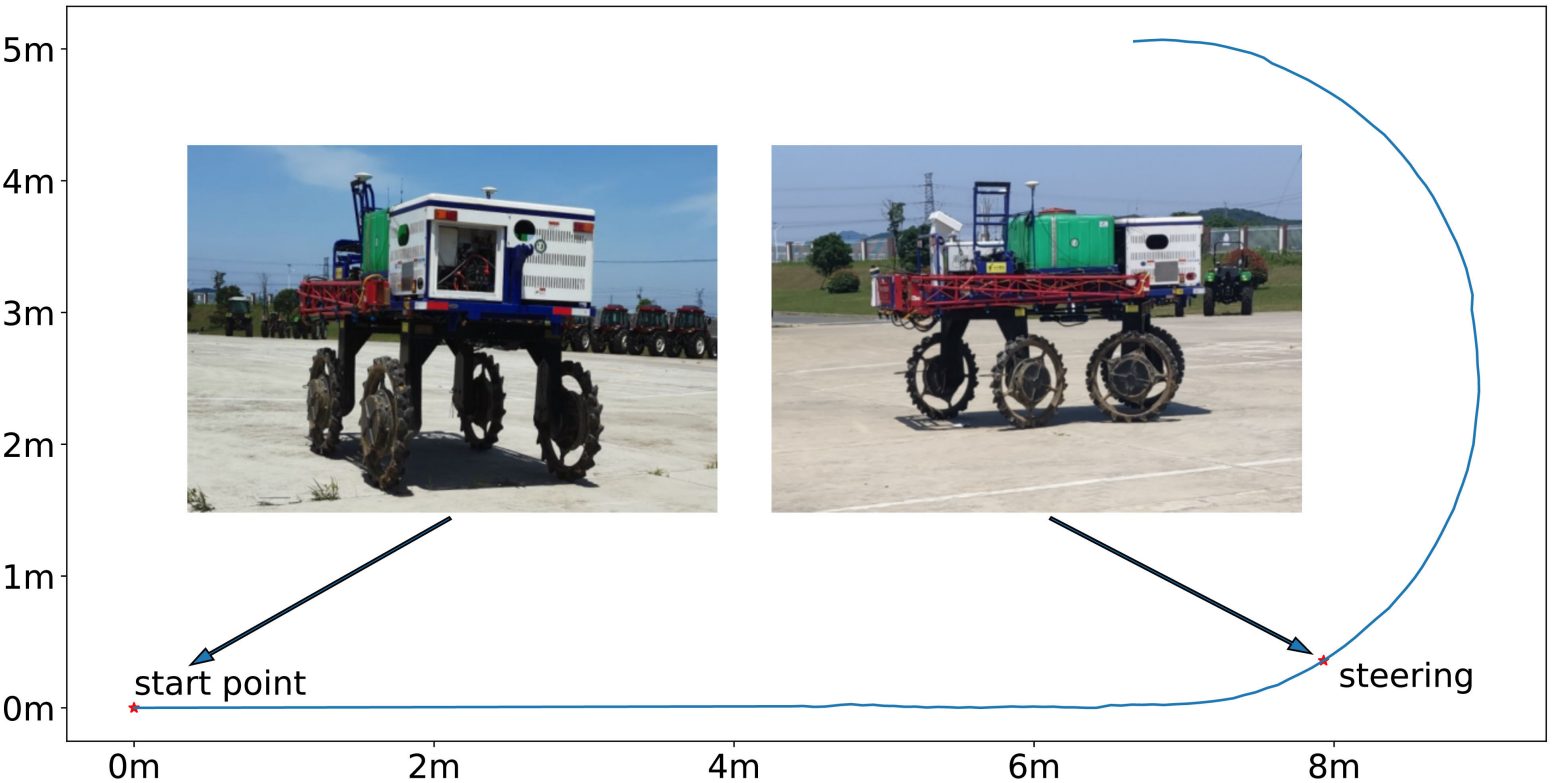
Speed and steering angle tracking test. The chassis holds still at the start point and steers after reaching the target speed.

This experiment aims to verify the control ability of the 4WSS chassis with SDC for speed and steering angle tracking. The experiment site is a flat concrete road with a length of 30 m and a width of 30 m. The test flow is shown in **Figure 11**: the line represents the chassis trajectory. The 4WSS held still, and the steering angle held 0 at the start point before operating. Then, target speed was set to an exact value. After the chassis was accelerated to the corresponding speed, the target steering angle was set to 24° because 24°, the program-limited steering angle, was the most frequently employed angle for making turns at field boundaries. Then, the chassis speed and steering control results can be achieved.

**Figure 12:**
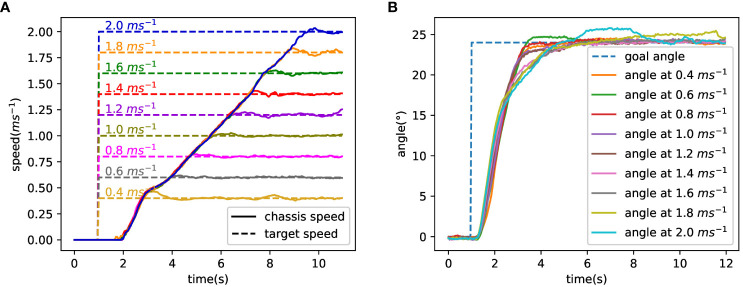
Test result of speed and steering angle. **(A)** Tracking curve at different target speeds. **(B)** The tracking curve of the front steering angle.

Referring to Equation 29, the maximum test speed is 2 ms^−1^. So, the 4WSS chassis test speed was set from 0.4 ms^−1^ to 2 ms^−1^ with an interval of 0.2 ms^−1^. The test results are shown in **Figure 12**, and their response characteristics are presented in **Table 1**. **Figure 12A** shows the tracking curve of the chassis speed at different target speeds. The dashed line is the target speed, and the solid line is the chassis speed (Equation 16 is used to calculate the 4WSS chassis speed using the four in-wheel motor speeds.). The target speeds vary from 0.4 ms^−1^ to 2 ms^−1^, with an interval of 0.2 ms^−1^. The rise times and overshoots of speed tracking control are shown in **Table 1**. It is obvious that as the speed increases, the rise time also increases linearly due to the acceleration that is set to 0.24 ms^−2^ for safety. When the chassis starts, the feedback speed is numerically 0 for nearly 1 s, while the actual value is increasing. Because the hall position sensors for the wheel motors are of low resolution, the driver cannot obtain effective speed feedback at very low speeds. When the velocity is 0.4 ms^−1^, the speed controller overshoots by 16.3%, whereas with 2.0 ms^−1^, it overshoots by only 1.7%. Because of the abnormal speed feedback, the wheel speed controller does not work correctly for the first 1 s. Therefore, the integral controller is integrated to a larger value, and then a more significant overshoot occurs. With a larger target speed, the acceleration time is longer and weakens such initial abnormal integration over time, resulting in a minor speed overshoot. And with different target speeds, the chassis speed can finally converge to the target speeds.

**Table 1:** Response characteristics of speed and steering angle.

Test speed (ms^−1^)	Speed		Angle	
	Rise time (s)	Overshoot (%)	Rise time (s)	Overshoot (%)
0.4	1.8	16.3	1.4	2.3
0.6	2.6	3.2	1.5	3.3
0.8	3.4	2.9	1.5	2.2
1.0	4.1	2.8	1.5	1.9
1.2	4.9	2.2	1.5	0.9
1.4	5.7	2.3	2.1	0.9
1.6	6.3	1.7	2.2	1.9
1.8	6.9	2.3	2.2	6.1
2.0	7.7	1.7	2.4	7.5

**Figure 12B** shows the tracking curve of the front steering angle during the turning test (The values of the front and rear steering angles are equal, so take one of them as an example to be analyzed.). Nine solid lines represent the track curves of the front steering angle at different speeds, with the dashed line representing the target angle. And the rise times and overshoots of steering angle tracking are also shown in **Table 1**. The figure shows that the dynamic and steady performance of the steering angle tracking varies at different target speeds. Obviously, the curves can be classified into three control performances based on goal speed values. When the goal speed is less than 1.4 ms^−1^, the rise times are less than 1.5 s and the overshoots are less than 4%. When the goal speed ranges over 1.4 ms^−1^~1.6 ms^−1^, a sudden increase of the rise times occurs that is over 2 s and the overshoots are still less than 4%. When the goal speed is above 1.6 ms^−1^, the rise times slightly increase and the overshoots are over 6%. The results indicate that as speed increases, angle control performance gradually declines. The ability to maintain differential speed is required at all times to track the goal steering angle. The in-wheel motor speed margin shrinks when the in-wheel motor speed gets closer to its maximum value. As a result, the performances of controlling the chassis differential speed and angle both decline gradually. Rewrite the maximum steering angular velocity *α*˙*_max_* in Equation 28 as Equation 37, and then *α*˙*_max_* can be used to describe the steering capability. *v_x,max_* represents the chassis speed. Consequently, it can be said that the steering ability gradually deteriorates as the angle and speed increase. This explains why its steering control ability gradually declined as speed increased.


(37) $${\dot{\alpha }}_{max}=2\frac{VL-\left|v_{x,max}\right|\left(L+D\left|sin\;\alpha \right|\right)}{DL}$$

**Figure 13:**
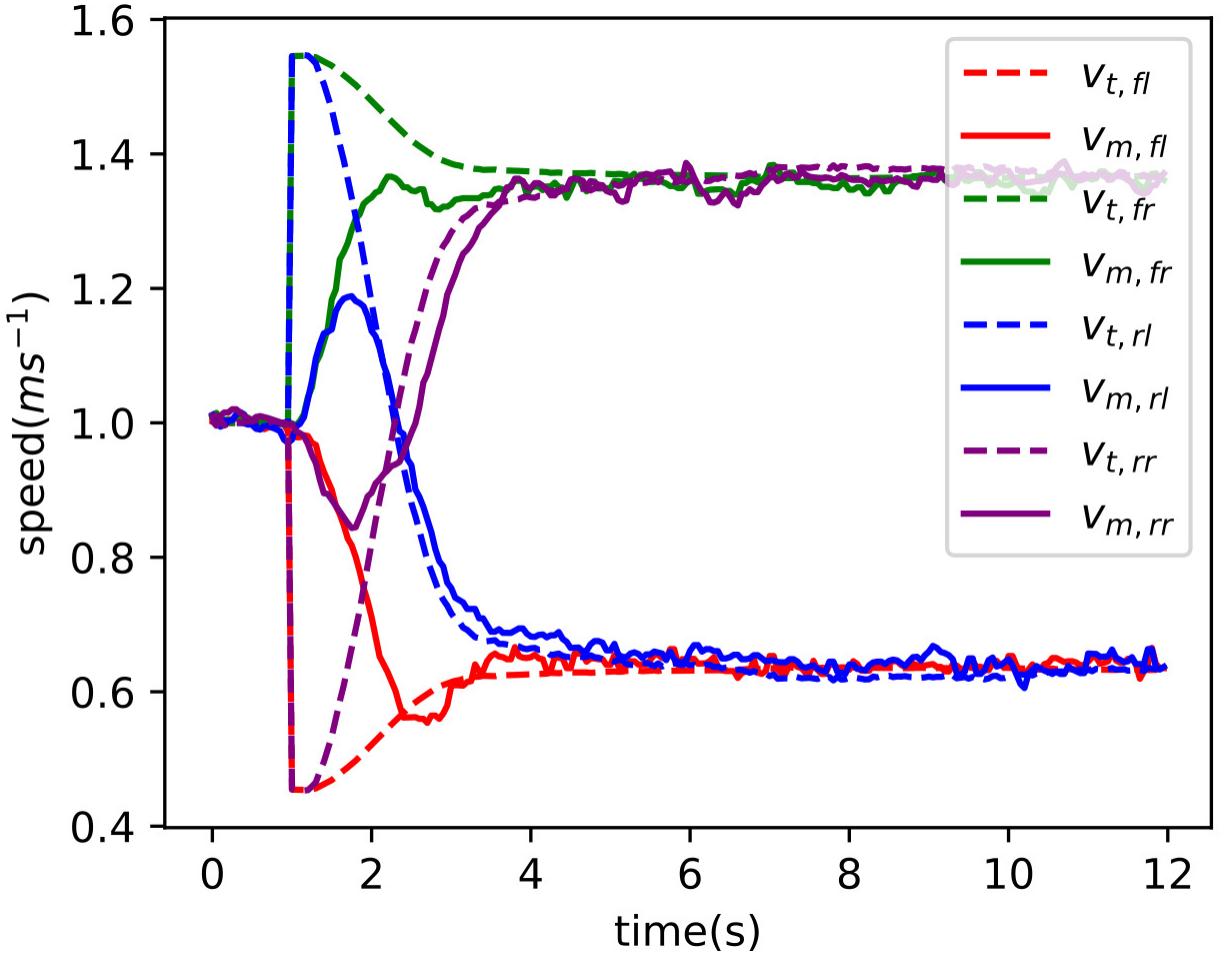
Four-wheel speed when steering at 1 ms^−1^.


**Figure 13** illustrates the individual wheel speeds while steering at a targeted speed of 1 ms^−1^. The target speed of four in-wheel motors is depicted by the dotted line, while the measured in-wheel motor speed is represented by the solid line. The various colors correspond to various motors. The figure shows that all four wheels have a speed of 1 ms^−1^ before steering. Then, the speeds of the *fr* and *rl* motors increase before decreasing to turn the chassis, while the speeds of the *fl* and *rr* decrease before increasing. After the transient process, the speeds of the right wheels converge to 1.4 ms^−1^, and the speeds of the left wheels converge to 0.6 ms^−1^. According to Equations 30–33, the target speeds of in-wheel motors are divided into two parts, as illustrated in Equations 38–40, named the kinematic part *S_inner_*,*S_outer_* and the *P* controller part *P_controller_*. The chassis speed is mainly the kinematic part *S_inner_* = *S_outer_* = *v_T_* = 1 ms^−1^ before steering. As the steering begins, the output of *P_controller_* controller part is a large value because of the maximum deviation of the steering angle and then decreases gradually with the convergence of the angle tracking. Finally, the outer and inner wheel speeds are *S_outer_* = 1.4 and *S_inner_* = 0.6 in order to maintain the differential steering.

(38) $$S_{inner}=v_{T}\;\left(1-\frac{D}{2I}\right)$$

(39) $$S_{outer}=v_{T}\;\left(1+\frac{D}{2I}\right)$$

(40) $$P_{controller}={\dot{\alpha }}_{p}\frac{D}{2}$$

### 3.2 Small turning radius

**Figure 14:**
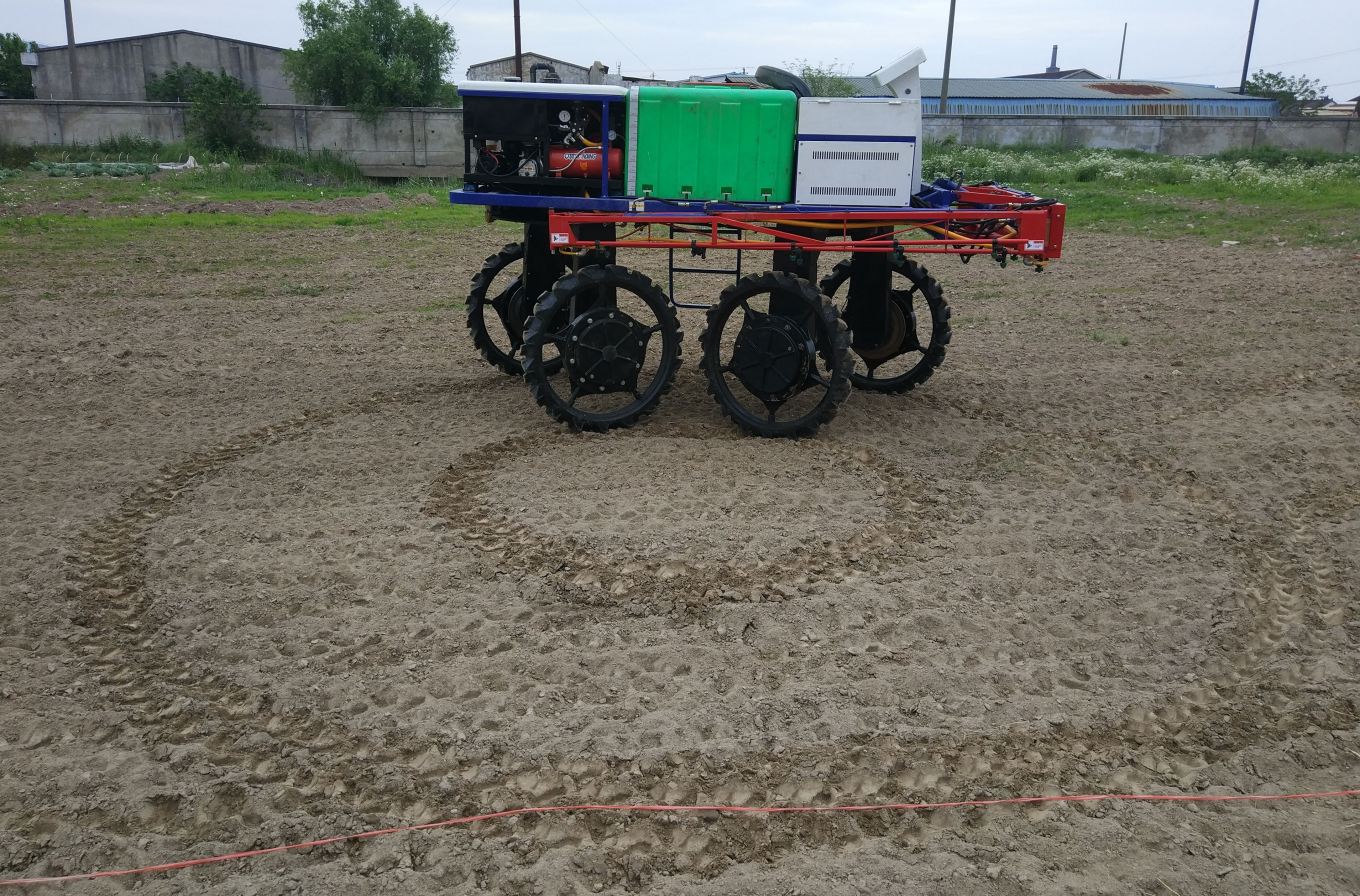
Turning trajectories of 24° steering angle.


Measurements of the 4WSS turning radius were performed at a speed of 0.8 ms^−1^ on a dry field. The 4WSS chassis formed two closed circular trajectories when it turned at a fixed steering angle, as shown in **Figure 14**. During the test, the chassis was located in the center of the test site. Then, the operator set the steering angle to 24° and drove the chassis to turn 360°. The chassis drew two circular trajectories on the ground. The formed two circles were measured, where the inner radius was 1,367 mm and the outer radius was 2,877 mm. So, the measured turning radius was 2,877 mm. From kinematics analyses, Equation 41 was obtained. *R*_1_ represented the theoretical radius for 4WSS chassis, and its result was determined to be 2,840 mm by substituting the parameter with the actual value. The error between the theoretical turning radius and the test result was within the allowable range.

(41) $$R_{1}=\frac{D}{2}+\frac{L}{2sin\left(\theta_{max}\right)}$$

Compared to other types of chassis, such as those by Wang et al. (2017) and Zeng et al. (2019). Their theoretical equation for the turning radius of the chassis was denoted by *R*_2_ and *R*_3_ in Equations 42 and 43. If their chassis parameters were indeed consistent with the 4WSS chassis (*L* = 1.7 m, *D* = 1.5 m), then their respective theoretical turning radius would be 4,759 mm and 4,874 mm. So, the turning radius of the 4WSS chassis was smaller.


(42) $$R_{2}=\frac{D}{2}+\frac{L}{sin\left(\alpha_{max}\right)}\left(1-0.5+0.5cos\left(\alpha_{max}\right)\right)$$

(43) $$R_{3}=L\sqrt{1+{\left(cot\left(\alpha_{max}\right)+\frac{D}{2L}\right)}^{2}}$$

### 3.3 Walking in a muddy field

To test the obstacle-surmounting capability of the chassis in the paddy field, three different test scenarios were set up, respectively: driving in a muddy field, driving across a deep gully, and driving over a ridge between field blocks. In order to be close to the actual working environment, the water load tank of the chassis was filled with 500 L of water during the test.

**Figure 15:**
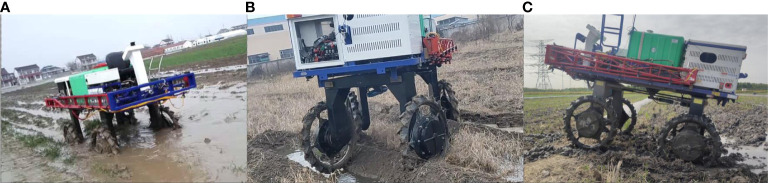
Field testing of the four-wheel self-steering (4WSS) chassis in a muddy field with three typical scenarios. **(A)** Driving in a muddy field. **(B)** Driving across a deep gully. **(C)** Driving over a ridge between field blocks.

The high-clearance chassis faced significant challenges in the muddy field environment. The soil in the field was soft and sticky, causing the chassis to frequently get stuck in the mud while driving, as illustrated in **Figure 15A**. During the muddy field test, the wheels sank to a depth of approximately 40 cm while moving, and the target speed ranged from 0.4 ms^−1^ to 1.8 ms^−1^. Despite these conditions, the 4WSS chassis exhibited smooth driving and steering capabilities.

As a kind of uncommon ground condition, the deep gully was inevitably encountered in the field. When traversing the deep gully, the chassis had to endure significant vibrations and heavy loads, especially when it was fully loaded. This posed a considerable challenge to both the structural integrity and power of the chassis. However, the 4WSS chassis offered a straightforward design without a transmission structure, making it less susceptible to damage. Additionally, the motor exhibited a robust overload capacity, enabling it to deliver substantial torque in a short time. The test was shown in **Figure 15B**. The speed of the test was set to 0.4 ms^−1^, and the deep gully in the field was over 20 cm wide and deeper than 40 cm. Crossing a deep gully horizontally was easy, as the front two wheels were subjected to the same forces and could be crossed simultaneously unless both wheels completely fell into the gully. Therefore, this test was chosen to go diagonally over a deep gully, working with the four wheels of the chassis plunging into the deep gully in turn and then climbing out.

To facilitate irrigation, the paddy field was usually divided into several blocks by ridges. So, the chassis needed to move across the field ridges when transferring between various fields. The test was shown in **Figure 15C**. The ridge was over 50 cm wide and 20 cm high, and the speed was set to 0.8 ms^−1^. The chassis moved stably across the ridge.

## 4 Conclusion

This paper proposes a four-wheel independent electric drive 4WSS chassis, in which the four wheels are fixed to the front and rear steering bridges, respectively. The chassis is controlled by the four-wheel differential speed to steer or move forward. The linkage is designed to constrain the front and rear steering angle, improving the stability of steering. Benefiting from the steering bridge differential design, it has a better obstacle-surmounting ability in extreme conditions such as a muddy terrain. A kinematic model was developed according to the structural characteristics of the chassis, and the mathematical relationships between the four-wheel speeds, chassis speed, steering angle, and steering angular velocity were obtained. Based on the kinematic model of the chassis, an SDC was built. In the SDC, the control references were the target speeds and target steering angles obtained from the remote control. The feedback signals were the current steering angle measured by position sensors mounted on the front and rear steering bridges. The desired speeds for the four wheels were determined as outputs by the SDC.

The speed tracking test revealed a maximum overshoot of 16.3% at a target speed of 0.4 ms^−1^. The steering angle tracking test showed that the performance of the steering angle control decreased as the speed rose. Therefore, the speed can approach 2.8 ms^−1^ when going straight but should be reduced to less than 2.0 ms^−1^ when turning at the maximum angle. Compared to other sprayer chassis, the 4WSS chassis has a smaller turning radius of 2,877 mm. And the chassis can move around with ease in conditions typical of the field, including muddy terrains, deep gullies, and ridges.

The 4WSS chassis is highly advantageous in the agricultural scene because it is capable of good passability at low speeds. Additionally, the chassis is designed closely in tandem with the future development direction of unmanned agricultural machinery. The advanced driving mode and steer-by-wire steering system eliminate the limitations associated with traditional agricultural machinery, showcasing significant potential in achieving an unmanned, intelligent, and information-driven chassis.

## Data availability statement

The original contributions presented in the study are included in the article/**Supplementary Material**, further inquiries can be directed to the corresponding author. The code for the 4WSS Sprayer is confidential, however the code for a small similar chassis model with SDC is available at https://gitee.com/he-siwei/fwied/tree/kinematics_v0.

## Author contributions

SH: Data curation, Investigation, Methodology, Software, Writing – original draft, Writing – review & editing. YS: Funding acquisition, Methodology, Supervision, Investigation, Conceptualization, Writing – review & editing. YZ: Data curation, Writing – original draft. HL: Supervision, Writing – review & editing.
